# Elementary Chemistry, Theoretical and Practical

**Published:** 1847-10

**Authors:** 


### PART III.—BIBLIOGRAPHICAL NOTICES.

### ARTICLE X.

*Elementary Chemistry, Theoretical and Practical.* By Geo. Fownes, Ph. D., Chemical Lecturer in the Middlesex Hospital Medical School, and to the Pharmaceutical Society of Great Britain. Edited with Additions by Robert Bridges, M. D., Professor of Chemistry in the Philadelphia College of Pharmacy, etc., etc. A new edition, with numerous illustrations. pp. 460, 12mo. Philadelphia. Lea & Blanchard. 1847. (From the publishers. For sale by Joseph Keen, Jr., Chicago.)

We have previously noticed the first American edition of this little work in terms of commendation and it gives us much pleasure to announce a second edition. It has been but two years since it first appeared in this country and the demand for it, which has brought out the present edition, alone proclaims sufficiently its merits. The American Editor has brought up the several subjects to the present date and thereby increased its value. We again most cheerfully recommend it as the best text book for students in attendance upon Chemical Lectures, we have yet examined. J. V. Z. B.

